# Theoretical Investigation of Proton Diffusion in Dion–Jacobson Layered Perovskite RbBiNb_2_O_7_

**DOI:** 10.3390/nano11081953

**Published:** 2021-07-29

**Authors:** Jing Shi, Chang Han, Haibo Niu, Youzhang Zhu, Sining Yun

**Affiliations:** 1 Department of Physics, Xi’an Jiaotong University City College, Xi’an 710018, China; jshi2002@163.com (J.S.); n_haibo@163.com (H.N.); yzh_zhu@xjtu.edu.cn (Y.Z.); 2 Qingdao Advanced Manufacturing Powder Engineering Research Center, Qingdao R&D Institute, Xi’an Jiaotong University, Qingdao 266330, China; han.ch@stu.xjtu.edu.cn; 3 MOE Key Laboratory for Nonequilibrium Synthesis and Modulation of Condensed Matter, School of Physics, Xi’an Jiaotong University, Xi’an 710049, China; 4 Functional Materials Laboratory (FML), School of Materials Science and Engineering, Xi’an University of Architecture and Technology, Xi’an 710055, China

**Keywords:** Dion–Jacobson, proton transport, SOFC, transition state, DFT calculations

## Abstract

Perovskite materials are considered to be promising electrolyte membrane candidates for electrochemical applications owing to their excellent proton- or oxide-ion-conducting properties. RbBiNb_2_O_7_ is a double-layered Dion–Jacobson perovskite oxide, with Pmc2_1_ symmetry. In this study, the electronic structure and proton-diffusion properties of bulk RbBiNb_2_O_7_ were systematically investigated using first-principles calculations. The unique layered crystal structure of RbBiNb_2_O_7_ plays a crucial role in proton storage and proton conductivity. Different proton-diffusion steps in RbBiNb_2_O_7_ were considered, and the activation energies of the relevant diffusion steps were evaluated using the climbing image-nudged elastic band (CI-NEB) technique. The proton diffusion in RbBiNb_2_O_7_ presents a two-dimensional layered characteristic in the a-b plane, owing to its layered crystalline nature. According to the transition state calculations, our results show that the bulk RbBiNb_2_O_7_ exhibits good proton-transport behavior in the a-b plane, which is better than many perovskite oxides, such as CaTiO_3_, CaZrO_3_, and SrZrO_3_. The proton diffusion in the Rb–O and Nb–O layers is isolated by a higher energy barrier of 0.86 eV. The strong octahedral tilting in RbBiNb_2_O_7_ would promote proton transport. Our study reveals the microscopic mechanisms of proton conductivity in Dion–Jacobson structured RbBiNb_2_O_7_, and provides theoretical evidence for its potential application as an electrolyte in solid oxide fuel cells (SOFCs).

## 1. Introduction

Perovskite-structured materials have attracted much attention owing to their extensive industrial applications, including solid oxide fuel cells (SOFCs), photovoltaics, catalysis, sensors, ferroelectrics, and memory storage [1,2,3,4,5,6]. An A-site or B-site doping strategy can significantly improve the performance of a perovskite material and expand its functionality [7,8,9,10,11,12]. A number of perovskite oxides have recently been studied as electrode materials in SOFCs [13]. These materials usually exhibit improved ionic conductivity performance at lower temperatures [14,15,16]. Under fuel cell operating conditions, the strongly correlated perovskite SmNiO_3_ undergoes a Mott transition from a metal into an insulator with high proton conductivity. The corresponding resistance of the H–SmNiO_3_ can be as low as 0.045 Ωcm^2^ at 500 °C [17]. When the H–SmNiO_3_ acts as an electrolyte in SOFCs, the open circuit voltage is 1.03 V, which is close to the theoretical value of the Nernst potential. The maximum power output reaches 225 mW/m^2^ at 500 °C [17,18].

In addition to ABO_3_ perovskite-structured materials, recent studies have focused on the high ionic conduction ability of layered perovskite materials with Dion–Jacobson, Ruddlesden–Popper, Aurivillius, and hexagonal perovskite structures [19,20,21,22,23]. These layered perovskite materials consist of two-dimensional (2D) perovskite octahedral layers in the a-b plane separated by cation layers in the c-direction. Their general chemical formulas can be expressed as $A\left(A_{n-1}^{’}B_{n}O_{3n+1}\right)$, $A_{2}\left(A_{n-1}^{’}B_{n}O_{3n-1}\right)$, $\left(Bi_{2}O_{2}\right)\left(A_{n-1}^{’}B_{n}O_{3n+1}\right)$, and $\left(A_{n}B_{n-1}O_{3n}\right),$ respectively [24,25]. B-site cations are usually transition metals located at the center of the octahedral, and form BO_6_. The BO_6_ networks are separated by A-site cations, and n is the number of BO_6_ octahedral layers (chemical formula $\left(A_{n-1}^{’}B_{n}O_{3n+1}\right)$) in the perovskite slab. In Dion–Jacobson structured materials, the A-sites can be occupied by alkali elements with +1 chemical valence, such as H, Na, K, Rb, and Cs cations. The A’ sites can be occupied by metal elements with +2 or +3 chemical valences, such as Ca, Sr, Ba, La, and Bi cations. B sites can be occupied by transition metal elements, such as Ti, Nb, and Ta cations, etc. [24,26,27]. Numerous studies have been conducted on Dion–Jacobson layered perovskites, based on their wide range of potential industrial applications, such as photocatalysis, solar cells, and ionic conductors [28,29,30,31,32]. When Dion–Jacobson perovskites are exposed to a hydrogen atmosphere, there is a dramatic increase in proton conductivity due to the high mobility of protons [33].

Recently, the Dion–Jacobson layered perovskite RbBiNb_2_O_7_ was synthesized by Li et al., through a conventional solid-state reaction [34]. At room temperature, RbBiNb_2_O_7_ has an orthorhombic structure with strong a^−^a^−^c^+^-type octahedral tilting in Glazer notation, which belongs to the Pmc2_1_ space group symmetry [25,28,34]. The lattice constants of the RbBiNb_2_O_7_ unit cell were measured as 5.463, 5.393, and 11.232 Å, respectively [34]. In RbBiNb_2_O_7_, the Nb cation forms NbO_6_ octahedra with neighboring oxygen ions. The NbO_6_ octahedra are connected to each other to form a double layer along the c-direction. Each double layer of NbO_6_ is separated by an Rb cation layer. The Rb cation layers combine with the ferroelectric off-centering displacements of A’-site Bi, and B-site Nb cations would introduce structural distortion and NbO_6_ octahedral tilting to the whole lattice. The Bi 6s lone-pair electrons and Nb 5d electrons have strong covalent interactions with the O 2p orbitals.

In this work, density functional theory (DFT) calculations are performed to study the proton-diffusion properties of the layered perovskite RbBiNb_2_O_7_. Two key issues are addressed. (1) The electronic structure of RbBiNb_2_O_7_ and its influence on the proton-binding behavior. (2) For proton diffusion in bulk RbBiNb_2_O_7_, various proton-transport steps, including inter-octahedral migration, intra-octahedral migration, and O–H reorientation are considered, and the corresponding activation energy barriers are evaluated by the climbing image-nudged elastic band (CI-NEB) calculations. The most stable proton-binding configurations are calculated with different oxygen environments, and the influence of the unique electronic structure of layered RbBiNb_2_O_7_ on proton binding is revealed. The distinctive two-dimensional layered proton diffusion in RbBiNb_2_O_7_ is discussed in detail. Furthermore, RbBiNb_2_O_7_ possesses strong a^−^a^−^c^+^-type octahedral tilting, which is one of the most important characteristics of Dion–Jacobson perovskites; therefore, the influence of strong octahedral tilting on proton diffusion is considered. To the best of our knowledge, this is the first systematic study of proton binding and two-dimensional proton diffusion in Dion–Jacobson RbBiNb_2_O_7_ perovskite, which provides a deep understanding of the advantages of layered perovskites as electrolytes in SOFCs.

## 2. Materials and Methods

All DFT calculations were performed using the density functional theory implemented in the Vienna Ab initio Simulation Package (VASP) [35,36]. Electronic wave functions were expanded in the plane wave functions with a kinetic energy cutoff of 400 eV. The initial structure information of bulk RbBiNb_2_O_7_ was obtained from our previous work [37]. A 2 × 2 × 1 RbBiNb_2_O_7_ supercell, including 88 atoms, was used in the calculations. The lattice constants a, b, and c are 10.728, 10.903, and 11.189 Å, respectively, which are consistent with the experimental values. The Brillouin zone was sampled by a 2 × 2 × 2 Monkhorst–Pack k-point mesh. The exchange–correlation interactions were described by the Perdew–Burke–Ernzerhof (PBE) functional in the framework of the generalized gradient approximation (GGA) [38]. The 4s, 4p, and 5s orbitals of Rb, 6s and 6p orbitals of Bi, 4p, 5s, and 4d orbitals of Nb, 2s and 2p orbitals of O, and 1s orbital of H are treated as valence orbitals. The convergence criterion was reached when the Hellmann–Feynman forces acting on each atom were less than 1 meV/Å. The VASPKIT code was used to process the density of states (DOS) and charge-density difference data [39]. Different proton-doped RbBiNb_2_O_7_ configurations were constructed and fully relaxed until the convergence criteria were reached. The climbing image-nudged elastic band (CI-NEB) method was used to determine the minimum-energy paths of proton migration and the corresponding activation energy barriers [40]. In order to study the charge transfer in the process of O–H bond formation, the charge-density difference was studied using the following formula: CHGCAR_Diff=CHGCAR_RbBiNb_2_O_7_/H-(CHGCAR_RbBiNb_2_O_7_+CHGCAR_H), where CHGCAR_Diff is the charge difference data, CHGCAR_RbBiNb_2_O_7_/H is the charge density of RbBiNb_2_O_7_ with doped H, CHGCAR_RbBiNb_2_O_7_ is the charge density of RbBiNb_2_O_7_, and CHGCAR_H is the charge density of H. The CHGCAR_Diff data was visualized by the VESTA code [41].

## 3. Results and Discussion

**Figure 1:**
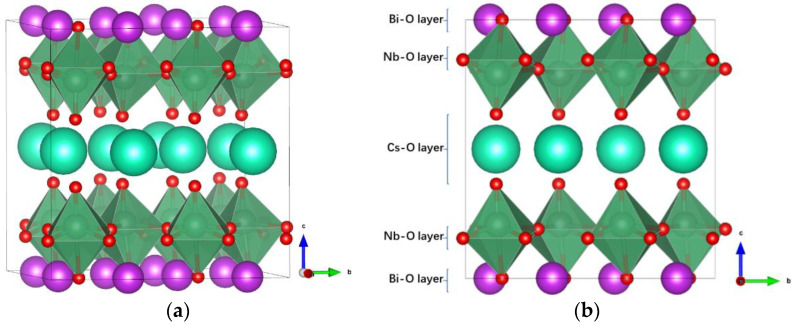
(**a**) The crystalline structure of RbBiNb_2_O_7_. (**b**) The RbBiNb_2_O_7_ lattice is divided into different layers.

Figure 1a shows the optimized crystal structure (space group: Pmc2_1_) of RbBiNb_2_O_7_. The large cyan spheres represent Rb ions, the purple spheres represent Bi ions, the dark cyan spheres represent Nb ions, and the small red spheres represent O ions. In RbBiNb_2_O_7_, the perovskite structure layer is two, thus two NbO_6_ octahedra are linked along the c-direction, with significant distortion and tilting. Owing to the layered crystal nature of RbBiNb_2_O_7_, it can be divided into Rb–O, Nb–O, and Bi–O layers along the c-direction, as shown in Figure 1b.

**Figure 2:**
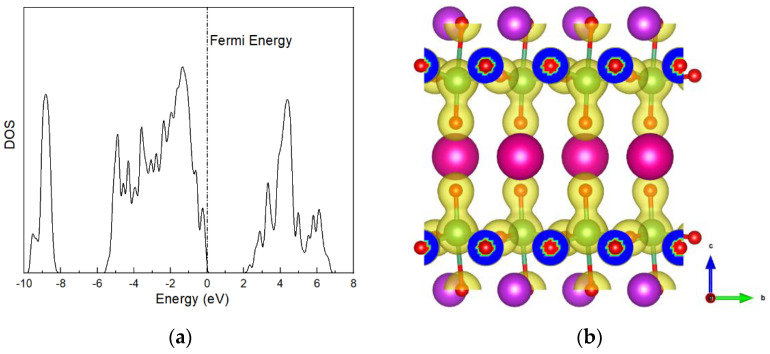
(**a**) Density of states and (**b**) charge density of RbBiNb_2_O_7_.

In SOFCs, the proton mobility is relevant to the electronic properties of electrolyte materials, which play a non-negligible role in the proton-binding properties and charge-transfer behaviors during proton diffusion. In order to reveal the intrinsic factors dominating the O–H binding and proton diffusivity in layered RbBiNb_2_O_7_, the total density of state (DOS) and charge-density diagrams are inspected and shown in Figure 2. It is worth noting that a high electronic density exists around the Fermi level (−1 eV, 0 eV), as shown in Figure 2a, implying a larger proton-storage ability in the layered RbBiNb_2_O_7_ [42]. In Figure 2b, the charge-density diagram shows that the charge density around the O ions located in the Rb–O layer is significantly stronger than those in the Nb–O and Bi–O layers.

In RbBiNb_2_O_7_, the chemical valences of Rb, Bi, and Nb cations are +1, +3, and +5, respectively. The Dion–Jacobson layered perovskite RbBiNb_2_O_7_ can be treated as a result of cutting the ABO_3_ perovskite structure along the (1 0 0) plane. The O ions located in the Rb–O layer are bonded with Rb and Nb, which leads to a distinct change in the bonding environment around these O ions. This feature can be studied using Bader charge analysis [43,44]. The Bader charge of O ions located in the Rb–O layer is −1.02 e, which is significantly lower than the charge of O ions located in the Nb–O and Bi–O layers (−1.21 e and −1.18 e, respectively). The higher charge density around the O ions located in the Rb–O layer proves that the bonding ability of these O ions is far from saturated, leading to the increased ability to form O–H bonds with protons in the Rb–O layer.

**Figure 3:**
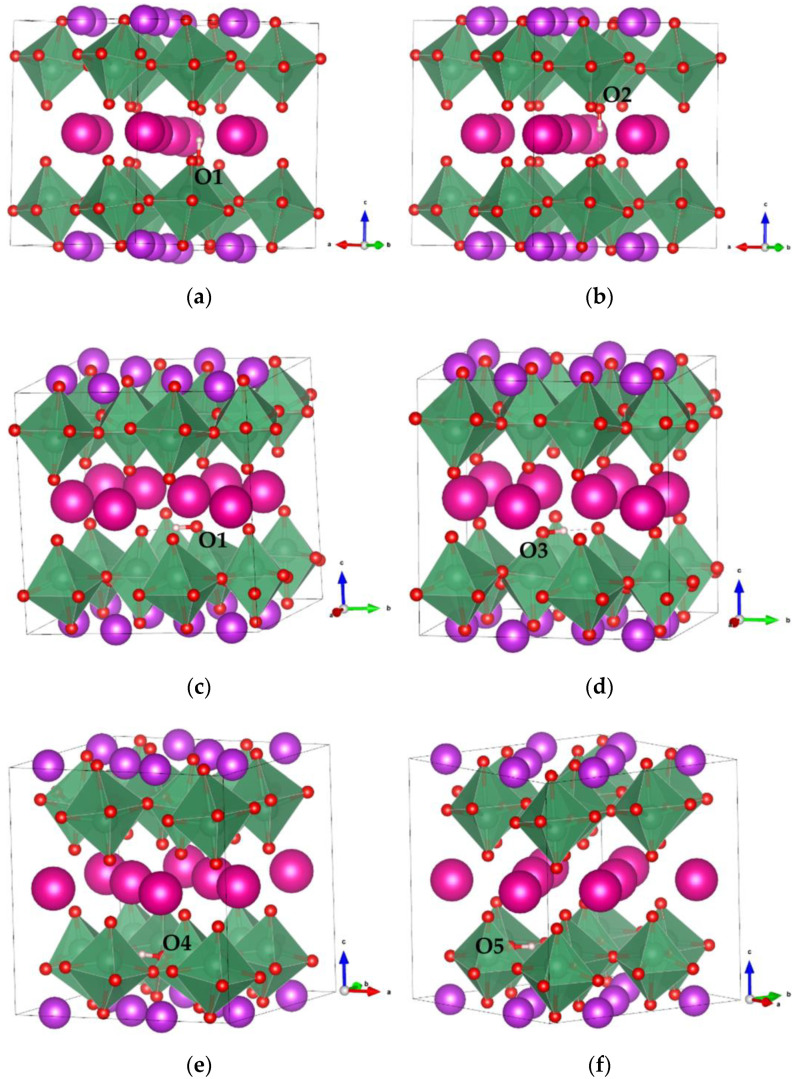
Proton binding at different O sites in RbBiNb_2_O_7_. (**a**,**b**) Proton binding with O1 and O2, respectively, with O–H bond perpendicular to the a-b plane. (**c**–**f**) Proton binding with O1, O3, O4 and O5, respectively, with O–H bond parallel to the a-b plane.

Before considering the proton-diffusion properties in RbBiNb_2_O_7_, we must determine the proton-binding configuration with the lowest energy. Here, one proton was introduced into the RbBiNb_2_O_7_ crystal structure, and different proton-binding configurations were considered, as shown in Figure 3. In these configurations, the proton binds to different O ions labeled as O1, O2, O3, O4, and O5. The order of structural stability of different proton-binding configurations is Figure 3a,b > Figure 3c,d > Figure 3e,f. It was found that the most stable O site for proton binding was around Rb, and the O–H bond direction was along the c-direction of the cell, which is attributed to the higher electron charge density and larger ion radii of Rb ions. The chemical valence of Rb is +1, and the larger ionic radii (1.61 Å) enlarged the space, which could decrease the electrostatic repulsion between the proton and Rb cations, and lower the total energy of the system [45]. Proton sites were also found to be the least energetically stable in the vicinity of a Bi ion because of the stronger Coulombic repulsion.

As RbBiNb_2_O_7_ has a strong ability to store protons, the bulk proton-diffusion mechanics in RbBiNb_2_O_7_ was studied, and the possibility of RbBiNb_2_O_7_ acting as an electrolyte membrane in SOFCs is discussed. It is well known that protonic conduction can be described by the Grötthuss diffusion mechanism, which involves two elementary mechanisms: the proton jumping from a bonded O ion to the adjacent O ions, and O–H rotation around the bound O ions by approximately 90° [14,46]. Therefore, long-range proton diffusion in ABO_3_ perovskites can be achieved by a sequence of proton migrations between neighboring O ions and the reorientations of O–H.

**Figure 4:**
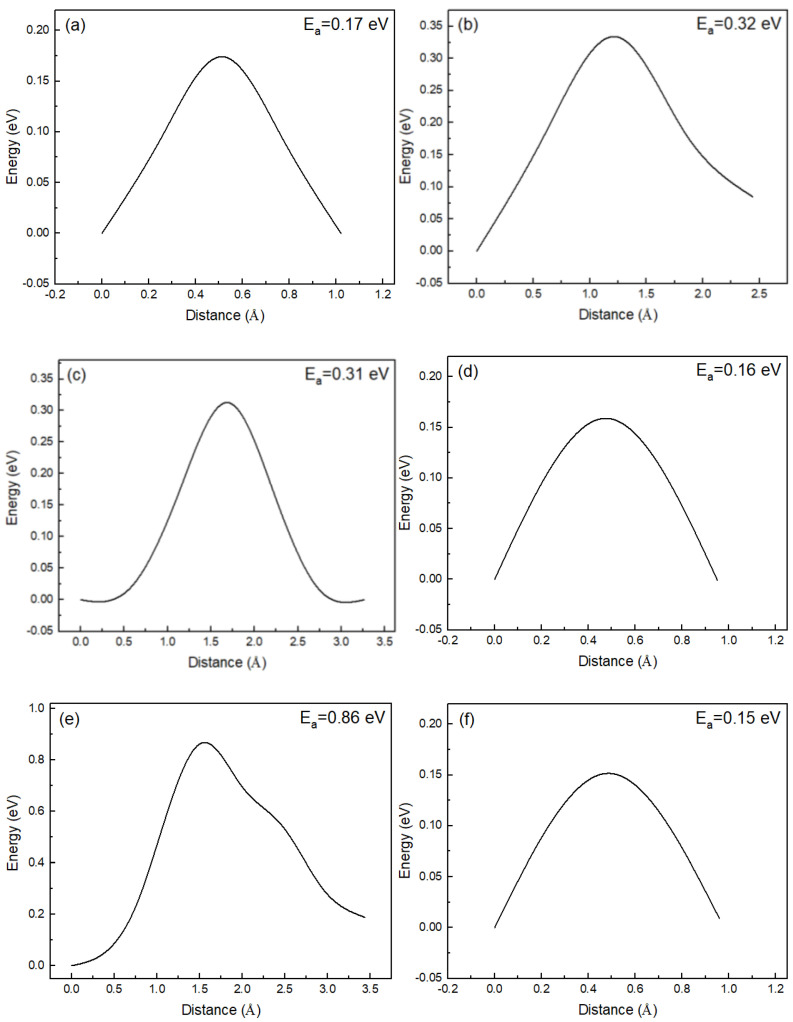
Transition states calculations of different proton-migration steps in RbBiNb_2_O_7_, (**a**) O1–O2, (**b**) first-type rotation of O1–H, (**c**) second-type rotation of O1–H, (**d**) O1–O3, (**e**) O3–O4, and (**f**) O4–O5.

In RbBiNb_2_O_7_, many different diffusion paths are possible depending on the distribution of O ions. These diffusion paths will contribute to the total proton flux in practice, but it is almost impossible to conduct a complete investigation of all the diffusion paths. Therefore, in this study, we focused on the most likely proton-migration steps in RbBiNb_2_O_7_. Based on previous studies, ionic transfer presents layered characteristics in layered perovskite materials, such as Dion–Jacobson or Ruddlesden–Popper perovskites [22,27]. In this work, different characteristic proton-transport steps (proton transfers in the Rb–O layer and Nb–O layer) were designed to evaluate the proton-diffusion properties in RbBiNb_2_O_7_, and the corresponding energy barriers are illustrated in Figure 4.

From the lowest-energy proton-binding configuration, it is convenient to transport protons from O1 to O2, with the corresponding migration energy barrier of 0.17 eV. When the proton binds at O1, the rotation of O–H can also occur. Because of the layered structure of RbBiNb_2_O_7_, there are two types of rotation of O–H that can take place at O1. The first type is O1–H rotation from along the c-direction to within the a-b plane; the second type is O1–H rotation on the a-b plane. For the first type of O–H rotation at O1, the energy barrier was calculated as 0.32 eV; for the second type, the energy barrier was 0.31 eV. These two types of O–H rotation at O1 present almost the same energy barriers.

After the first type of O1–H rotation, the O–H direction is parallel to the a-b plane, and the octahedral tilting of the corresponding NbO_6_ changes significantly, as shown in Figure 3a,c. The change in octahedral tilting decreases the distance between O1–adjacent O ions (i.e., O1 and O3 in Figure 3). The distance between O1 and O3 is decreased from 3.56 Å to 2.70 Å after the O–H rotation, which would significantly facilitate the proton migration between O1 and O3. The activation energy barrier of proton transport from O1 to O3 is 0.15 eV, which is significantly lower than that of the O–H rotation. Ultimately, the long-range proton-diffusion pathways in the Rb–O layer of RbBiNb_2_O_7_ can be achieved through the intra-octahedral migration of protons combined with O-H rotation.

The proton can also be transported to the Nb–O layer of RbBiNb_2_O_7_ (Figure 3e,f), through the inter-octahedral transport steps. The energy barrier of proton transport from the Rb–O layer to the Nb–O layer is 0.86 eV, as shown in Figure 4e. This energy barrier is significantly higher than the proton-migration energy barrier in the Rb–O layer, which means that proton transport from the Rb–O layer to the Nb–O layer is isolated. Therefore, proton diffusion shows a layered characteristic in RbBiNb_2_O_7_, and the proton transport between different layers is unfavorable. In Figure 4e, a sub-step is observed with an energy barrier of 0.37 eV, which corresponds to the rotation of O–H at O4. The O–H direction changes from along the c-direction to parallel to the a-b plane, while the proton is located in the Nb–O layer. In the Nb–O layer, intra-octahedral proton transport is favored because of the strong octahedral tilting behavior in RbBiNb_2_O_7_. The nearest O ions (O4 and O5) between adjacent octahedra are approximately 2.71 Å. The energy barrier of the intra-octahedral proton transport is 0.15 eV, as shown in Figure 4f.

The calculated energy barriers of proton migration in bulk RbBiNb_2_O_7_ are within the range of many studied proton-conducting materials. The energy barriers of proton jumping (~0.16 eV) in bulk RbBiNb_2_O_7_ are significantly lower than many studied perovskite oxides, such as: CaMnO_3_ (1.76 eV), CaTiO_3_ (1.68 eV), CaZrO_3_ (2.51 eV), and SrZrO_3_ (0.80 eV) [46]. The energy barriers of O–H rotation (~0.32 eV) in bulk RbBiNb_2_O_7_ are lower than A-site Na-doped BaZrO_3_ (0.54 eV) [11], and can be comparable with B-site Y- and Gd-doped BaZrO_3_ (0.32 eV and 0.34 eV, respectively) [47]. The proton-migration energy barriers in bulk RbBiNb_2_O_7_ demonstrate improved proton-conduction properties with potential applications as electrolytes in SOFCs.

**Figure 5:**
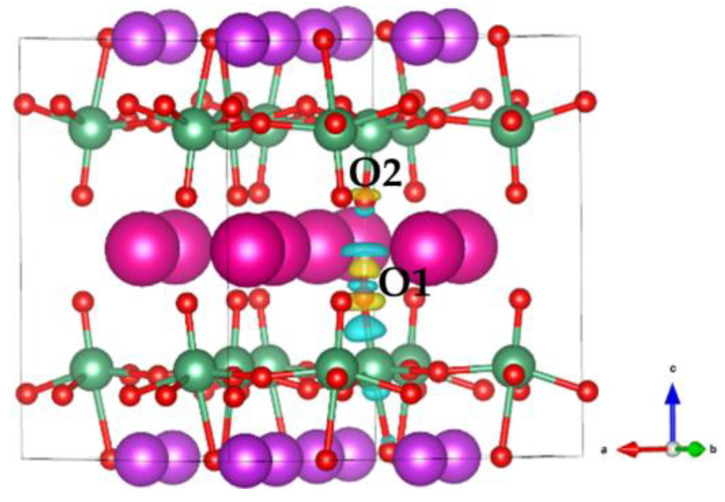
Charge transfer behavior when proton binds at O1.

The charge density difference of the most stable proton-doped RbBiNb_2_O_7_ configuration is shown in Figure 5. It worth noting that the charge density changed significantly after the proton was introduced. A significant weakness of the Nb–O1 bond was observed, along with the formation of the O1–H bond. The electron charges were depleted between the Nb–O1 bond, and accumulated between the O1–H bond, indicating that the charge transfer from the Nb–O1 bond to the O1–H bond. The Bader charge of the proton is +0.68 e, indicating the effective charge transfer between the proton and O1. In Figure 5, the charge distribution of O2 also changed, which indicates the formation of a hydrogen bond between the proton and O2. The hydrogen bond that formed between O1–H and the adjacent O2 ions would promote proton diffusion.

Owing to the limitations of density functional theory (DFT), the effects of temperature were not considered. However, in a real-world situation, the temperature is important for proton diffusion. At low temperatures, lattice vibrations are frozen out, and proton diffusion is achieved via the zero-phonon tunneling mechanism [4,48,49]. In this mechanism, the proton-hopping rate is weakly dependent on the temperature, which is different from the thermally activated proton-diffusion behavior. At higher temperatures, in which many phonons of the lattice are excited, proton diffusion is dominated by phonon-assisted hopping [50,51]. In this mechanism, proton migration is strongly correlated with the thermal vibrations of the lattice. Furthermore, at higher temperatures, thermal fluctuations of the lattice can occasionally produce configurations with energy barriers that are easily overcome by protons, thus promoting proton diffusion [4,52].

## 4. Conclusions

The proton-diffusion properties in Dion–Jacobson layered perovskite RbBiNb_2_O_7_ were theoretically studied through DFT computations. After a proton was introduced into the bulk RbBiNb_2_O_7_ structure, a O–H bond was formed, and the corresponding octahedral tilting significantly changed. This led to the reduction in the distance between adjacent O ions and the formation of a hydrogen bond, which promoted proton migration between the two adjacent O ions. For different proton diffusion steps in the a-b plane, the activation energy barriers were almost the same, with values of approximately 0.16 eV. The proton diffusion in RbBiNb_2_O_7_ showed a layered diffusion characteristic. The proton diffusion in the Rb–O layer and Nb–O layer was isolated by a 0.86 eV energy barrier. Based on our study, two important factors controlling the proton diffusion in the bulk RbBiNb_2_O_7_ were revealed. One is that the unsaturated bonding environment around the O ions in the Rb–O layer, which provides the high ability for proton storage in RbBiNb_2_O_7_. The other is that two-dimensional long-range proton-diffusion paths are achieved in layered RbBiNb_2_O_7_. Therefore, the proper activation energy barriers of proton transport in the Rb–O layer and the high phase stability make RbBiNb_2_O_7_ a potential high-performance proton-conducting electrolyte material for SOFCs.

## Data Availability

The data presented in this study are available on request from the corresponding author.
